# Prevention and Management of Postoperative Ileus: A Review of Current Practice

**DOI:** 10.7759/cureus.22652

**Published:** 2022-02-27

**Authors:** Zeeshan H Khawaja, Ahmed Gendia, Naqqash Adnan, Jamil Ahmed

**Affiliations:** 1 Department of Surgery, Wrightington, Wigan and Leigh NHS Foundation Trust, Wigan, GBR; 2 General and Colorectal Surgery, Northampton General Hospital, Northampton, GBR; 3 Colorectal and General Surgery Department, Northampton General Hospital, Northampton, GBR

**Keywords:** laparoscopic surgery, robotic surgical procedures, gastrointestinal obstruction, enhanced recovery after surgery, postoperative ileus

## Abstract

Postoperative ileus (POI) has long been a challenging clinical problem for both patients and healthcare physicians alike. Although a standardized definition does not exist, it generally includes symptoms of intolerance to diet, lack of passing stool, abdominal distension, or flatus. Not only does prolonged POI increase patient discomfort and morbidity, but it is possibly the single most important factor that results in prolongation of the length of hospital stay with a significant deleterious effect on healthcare costs in surgical patients. Determining the exact pathogenesis of POI is difficult to achieve; however, it can be conceptually divided into patient-related and operative factors, which can further be broadly classified as neurogenic, inflammatory, hormonal, and pharmacological mechanisms.

Different strategies have been introduced aimed at improving the quality of perioperative care by reducing perioperative morbidity and length of stay, which include Enhanced Recovery After Surgery (ERAS) protocols, minimally invasive surgical approaches, and the use of specific pharmaceutical therapies. Recent studies have shown that the ERAS pathway and laparoscopic approach are generally effective in reducing patient morbidity with early return of gut function. Out of many studies on pharmacological agents over the recent years, alvimopan has shown the most promising results. However, due to its potential complications and cost, its clinical use is limited. Therefore, this article aimed to review the pathophysiology of POI and explore recent advances in treatment modalities and prevention of postoperative ileus.

## Introduction and background

Postoperative ileus (POI) is considered as intolerance of oral intake due to disruption of the normal coordinated propulsive motor activity of the gastrointestinal (GI) tract following abdominal or non-abdominal surgery, without any mechanical element [1-3]. Multiple studies have given various definitions for POI, with its occurrence ranging from postoperative day 3 to greater than postoperative day 7. Yet in these studies, the condensed definition of POI generally included symptoms of intolerance to diet, lack of passing stool, abdominal distension, or flatus [4].

The absence of accurate classification criteria makes it difficult to determine the exact incidence. However, a study conducted in the United States reported an incidence of 17.4% with a sample size of approximately 17,000 colectomies [5]. Up to one in eight patients of gastrointestinal surgery is affected by prolonged postoperative ileus, leading to the discomfort of patients and prolonged hospital stay [6]. Furthermore, demographical characteristics such as male sex, increasing age, and prior abdominal surgeries have all been found to be factors associated with POI [4].

It is sometimes referred to as type 1 intestinal failure and can be classified as primary or secondary, depending on whether it has developed in the presence of a known precipitating factor or not. This usually occurs after surgery without any mechanical factors that can disrupt the normal synchronized motor activity of the digestive tract [7].

Some of the most common causes of secondary POI include wound infections, intra-abdominal collections, anastomotic leaks, or other sources of sepsis. Some studies suggest that the normal intestinal motility (IM) should be restored completely within two to three days after surgery [7]. Hence, there is a general consensus that some extent of POI is a normal physiological effect as a consequence of abdominal surgery [8,9]. The type of surgery being performed, patient’s factors, and intraoperative complications need to be considered when differentiating physiological and pathological triggers for POI. Physiological POI occurring after the surgery is benign and resolves without any intervention. However, if the ileus persists, the patient is diagnosed with having "prolonged" or "pathologic" POI. Although pathologic POI is still poorly defined, it is considered when post-surgical gut recovery is prolonged for more than three to seven days [6].

Regardless of the type, POI is well known to have a critical effect on patient morbidity due to symptoms including pain, vomiting, abdominal distension, poor oral feeding, nausea, and lack of defecation or passing flatus [7]. Despite recent advances in perioperative care and surgical techniques, POI remains one of the most frequently encountered challenges after surgery [10,11]. It is possibly the single most important factor to prolong the length of stay (LOS) after bowel surgery with a significant deleterious effect on healthcare costs in surgical patients [12]. The average weekly costs for excess bed days for non-elective and elective inpatients are £2,089 and £2,532, respectively; hence, prolonged stay due to POI can have a significant negative economic impact on health care [13]. 

This article aims to review the current knowledge about the pathophysiology and more importantly the treatment of POI. The roles of Enhanced Recovery After Surgery (ERAS) protocols, minimally invasive surgical approaches, and various pharmaceutical agents aimed at minimizing the incidence and duration of POI in current surgical practice are appraised.

## Review

Pathophysiology

The motility of the gut is maintained by a complex interplay between the central and enteric nervous systems as well as hormonal and local factors acting directly on the intestinal smooth muscle [7,14]. Control of gastrointestinal motility is the interplay between many factors, which has made it difficult to determine the exact pathogenesis of POI. However, it is hypothesized to be due to an alteration to one of the factors that regulate bowel motility. There are four distinct mechanisms described in the literature, and an abnormality in any of the mechanisms due to surgical trauma or bowel manipulation can ultimately lead to POI. The four pathways can be broadly classified as neurogenic, inflammatory, hormonal, and pharmacological [15].

Neural

The motility of the GI tract is controlled by close coordination between the sympathetic, parasympathetic, and enteric nervous systems. The parasympathetic nervous system has a stimulatory effect on motility, whereas the sympathetic nervous system has an inhibitory effect. Surgical incision and penetration of internal viscera have been shown to activate the sympathetic response, leading to decreased gut motility by inhibiting the normal motilin motor complex (MMC) system [15].

Inflammatory and hormonal

Manipulation of the viscera at the time of surgery leads to the activation of macrophages and other inflammatory cells in the muscularis externa. This, in turn, leads to the release of pro-inflammatory cytokines and chemokines, which mediate inflammation and attract more cells. Many mediators, particularly nitric oxide and prostaglandins, inhibit smooth muscle motility and contribute to ileus [16]. This hypothesis has been supported by studies conducted in rats in which varying degrees of penetration led to a varying amount of cytokine release [17]. Several local mediators and hormones are attributed to this effect rather than a single cause, yet studies have reported nitric oxide (NO) to be the major inhibitory noradrenergic noncholinergic neurotransmitter of the GI tract, which may cause POI [18].

Pharmacological

Pharmacological agents, most commonly anesthetics and opioids, have been known to cause and prolong POI [19,20]. Anesthetics reduce motility by stabilizing neural membranes; therefore, they have the greatest effect on areas that rely heavily on neural integration. The colon lacks gap junctions as opposed to other parts of the gut; therefore, it is particularly susceptible to the action of these agents [7]. Similarly, opioids such as morphine are commonly used to reduce post-surgical pain, and they are known to mediate their actions via interaction with μ receptors in the GI tract [15]. Opioids have been associated with POI by decreasing gut motility, acetylcholine release, and delaying gastric emptying.

How can we minimize the incidence of postoperative ileus?

Different strategies have been introduced over time in surgical practice, which aimed at improving the quality of perioperative care by reducing perioperative morbidity in the hospital. Examples of these strategies include ERAS protocols, minimally invasive surgical approaches, and the use of specific pharmaceutical therapies [21]. Currently, there is enough evidence to suggest that these advances in surgical techniques have been shown to minimize surgical stress response and help to maintain normal physiology throughout the perioperative period. This, in turn, ultimately results in the prevention or curtailing of postoperative ileus.

ERAS protocols or enhanced recovery programs (ERPs)

The change in surgical practice recently has resulted in the earlier restoration of postoperative physiological function with resultant improved outcomes and shortened hospital stay. ERAS or ERP pathways are a "multimodal rehabilitation" program that has shown to improve surgical outcomes and patient satisfaction after elective surgery [17,22,23]. Initiated in the early 1990s, the ERAS pathways comprise a series of measures that are implemented before, during, and after surgery, with the intention that their cumulative effect will accelerate postoperative recovery, reduce complications and lead to an early return in daily activities [24]. Initially, ERAS protocols were extensively used for colorectal surgeries primarily; however, over time, other surgical specialties have adopted the protocol as well [12,14,25].

ERAS pathways can be divided into preoperative theater day, intraoperative, and postoperative interventions, all of which attempt to bring early gut function, thus promoting early postoperative recovery [26]. For example, epidural analgesia is thought to increase the motility of the gut by causing a relative parasympathetic overdrive. Similarly, other components of ERAS such as avoidance of opiates, use of prokinetic agents, and avoidance of nasogastric tubes postoperatively promote early recovery of gut function [26].

Some studies have reported a lower incidence of POI when using ERAS protocols in comparison to conventional therapy, while others have reported comparable rates of POI in both methods [27-29]. Zeng et al. showed that ERAS, in comparison to conventional protocols, had a shorter time to first flatus (two versus three days, p < 0.001), time to first stool (three versus four days, p = 0.001), and time to oral intake (one versus four days, p < 0.001); thus, ERAS comparatively had a faster return of gastrointestinal function postoperatively [30]. (This study was done in 2017.) Similarly, other studies looking at ERAS alone have also shown it to be associated with faster bowel recovery, with decreased time to passage of flatus, the passage of stool, and tolerance to oral feed without increasing admission rates [26,30-32]. Early studies assessing its success in colorectal surgery reported a 50% reduction in complications and 2.5 days of reduced LOS on average [33]. Interestingly, Kennedy et al. found that patients taking medicines under the ERAS protocol required fewer narcotics and also had a shorter LOS [34]. Apart from reduced morbidity, ERAS pathways may aid in reducing the burden on the healthcare economy, with savings ranging from approximately $2,245 to $7,600 per patient as estimated by a study [26].

An important component of ERAS is fluid management, to avoid fluid shifts and minimize complications during surgery. Fluid management focuses on preoperative, intraoperative, and postoperative regulation of fluids [24]. Preoperative guidelines recommend patients to continue clear carbohydrate-rich fluids until two hours before induction of anesthesia [35]. Intraoperative fluid therapy aims at achieving adequate perfusion of organs with adequate end circulating volume. Similarly, it is recommended to start an oral diet and fluids soon after surgery and minimize fluids postoperatively as it has been shown to be associated with faster bowel recovery and shorter LOS [36,37]. It is established that both hypovolemia and hypervolemia are associated with complications. Hypovolemia can lead to decreased organ perfusion, sepsis, and multi-organ failure, whereas hypervolemia can contribute to edema and has shown to also increase the incidence of POI [36].

Gum chewing, which is another component of ERAS, has been used commonly for early recovery from POI as it leads to vagal stimulation and therefore increases gastrointestinal secretions, increases gut motility, and reduces inhibitory sympathetic signals [38]. Furthermore, it increases salivary and pancreatic secretions, and some sugars found in sugar-free gum have been reported to reduce postoperative ileus [39,40]. A study postulated that chewing gum alone could save the US healthcare system around $118,828,000 in hospital stay costs [41]. It has been shown to expedite the return of gut function postoperatively in several randomized trials and at least three meta-analyses [42-44].

Minimally invasive surgical approach

Minimally invasive surgical procedures have gained a lot of popularity in the past few years, having a significant impact on surgical practice for both surgeons and patients alike. Laparoscopic techniques have been shown to involve less tissue manipulation, reduced surgical stress and trauma, and earlier patient recovery [21,33]. Hence, it has attracted most surgical specialties to use it quite enthusiastically and has become a standard approach for several surgical procedures.

Laparoscopic techniques are considered to reduce the severity and duration of ileus, resulting in shorter hospital LOS [22]. In comparison to open surgery, numerous articles have shown that minimally invasive surgical procedures, including laparoscopy, are associated with fewer postoperative complications including POI [45-47]. Randomized controlled studies have also demonstrated a laparoscopic approach in being more superior for reducing the duration of POI after abdominal surgery [48-50]. Recently, a randomized clinical trial (LAFA study) showed that laparoscopy in combination with fast-track multimodal management is the best perioperative strategy in patients undergoing colonic surgery [51]. Similarly, a systematic review by Spanjersberg et al. concluded that morbidity (including POI) and hospital stay were reduced when both laparoscopy and ERAS are combined [52]. Nonetheless, laparoscopy still plays an important role in reducing the incidence of POI and shares the same goals as ERAS without compromising other outcomes [53].

While prior studies quoted the benefit of laparoscopic surgeries, several studies have shown contrasting results. A prospective study of 80 participants reported that laparoscopic resection was not associated with a reduction in the duration of ileus or hospital stay in patients undergoing colorectal resection. However, the small sample size of the study might have been a limitation affecting the results [54]. Similarly, other studies have reported no difference in the incidence of POI if minimally invasive surgery is used instead of open surgery [55,56]. In a recent clinical trial investigating the impact of the laparoscopic approach using the ERAS protocol, Basse et al. failed to show any advantage of the laparoscopic approach in colonic resections [57].

Looking at the minimally invasive surgeries, in particular, several studies have also compared the efficacy of different types of minimally invasive procedures among themselves, particularly robotic surgery versus laparoscopic surgery. Solaini et al. compared several postoperative complications including postoperative hemorrhage, ileus, incisional hernia, infection, and abdominal abscess and found that there was no difference between laparoscopic surgery and robotic surgery [58]. On the contrary, a network meta-analysis found that the ileus rate and LOS were significantly lower with the robotic-assisted surgery group as compared to laparoscopic surgery and open surgery [59].

Pharmacological therapies

There are several pharmacological therapies, including carbohydrate loading, use of non-steroidal anti-inflammatory drugs (NSAIDs), prophylactic anti-emetics, and epidural and regional analgesia, all of which are an integral part of the ERAS pathways. These therapeutic modalities have been well known to have a significant impact on the early return of gut function as shown in a review by Kehlet et al. [60].

Pharmacological therapies are still evolving and have been extensively investigated over the recent years as they can play a principal role in the prevention and management of POI. Among these drugs, alvimopan, which is an oral peripherally acting mu-opioid receptor antagonist, has shown the most promising results. However, the US Food and Drug Administration (FDA) has limited the indications for drug use due to increased risk for neoplastic or cardiovascular complications [61]. It is more specifically indicated for hastening gastrointestinal recovery following partial bowel resection along with primary anastomosis. Furthermore, it is limited to inpatient care at hospitals that meet the standards of evaluating outcomes and staff training. Its routine clinical usage may further be infrequent due to the high cost of the drug [62]. Mechanisms of action of recent pharmacological agents for the treatment of POI are given in Table 1, and findings of recent studies regarding the use of several pharmacological agents and their association with POI are given in Table 2.

**Table 1:** Summary of the mechanism of action of recent pharmacological therapies used for the treatment of postoperative ileus GI: Gastrointestinal; POI: Postoperative ileus.

Pharmacological Therapy	Mechanism of Action (MOA)
Alvimopan (μ-opioid receptor antagonists)	Studies have identified several opioid receptors most notably mu, delta, and kappa. Opioids reduce GI motility by acting on these receptors and thus provide more time for water and electrolyte absorption and are effective as anti-diarrheal. μ-opioid receptor antagonists will block the μ opioid receptor and thus improve gut motility.
Prucalopride (serotonin receptor-5HT4 agonists)	Prucalopride is a highly selective, high-affinity 5-HT4 receptor agonist that binds to 5-HT4 receptors on enteric neurons, thus facilitating cholinergic, nonadrenergic, and non-cholinergic neurotransmission.
Neostigmine (cholinergic agonist)	Acetylcholine is a neurotransmitter released at synapses and is responsible for initiating muscle contraction in the gut wall. The hormone is inhibited by the enzyme acetylcholinesterase. Neostigmine is a reversible inhibitor of acetylcholinesterase and has been proven to be successful in POI management.
Calcitonin gene-related peptide (CGRP) receptor antagonist	CGRP is released from myenteric nerves and activates resident leukocytes and thus contributes to POI. CGRP receptor antagonist blocks the CGRP receptor and improves gut motility.
5-HT3 receptor antagonists	The 5-HT3 receptor is found on macrophages in the GI tract. The 5-HT3 receptor antagonists reduce intestinal motility-induced infiltration of inflammatory CD68-positive macrophages and myeloperoxidase-stained neutrophils. The anti-inflammatory action is attributed to improving delayed GI transit.
Lidocaine	Lidocaine has shown to suppress the inflammatory reaction as visualized by potent inhibition of plasma extravasation in the obstructed gut. Net fluid secretion is reversed, and inflammation in the obstructed bowel has also been shown to reduce.
Prokinetic agents (metoclopramide and erythromycin)	Metoclopramide has been reported to act as a cholinergic agonist and dopaminergic antagonist. Erythromycin acts as a motilin receptor agonist and stimulates the release of migrating motor complexes (MMCs).

**Table 2:** Summary of recent studies on pharmacological therapies used for the treatment of postoperative ileus GI: Gastrointestinal; POI: Postoperative ileus; DOCIVA: Decrease opioid consumption with intravenous (IV) acetaminophen after colorectal surgery; SQ: Subcutaneous.

Author	Year	Drug	Study Setting	Population	Outcomes
Drake et al. [63]	2016	μ-opioid receptor antagonists, serotonin receptor agonists, and ghrelin receptor agonists	Systematic review	5,836 patients from 17 studies	The use of μ-opioid receptor antagonists significantly reduces bowel recovery time following major abdominal surgery. The use of serotonin receptor agonists may also be of use; however, there is a lack of high-quality homogenous trials to support this. There is good evidence to suggest that ghrelin receptor agonists are not useful for the prevention of postoperative ileus.
Liang-Xu et al. [64]	2016	Alvimopan	Systematic review and meta-analysis	4,075 patients from 9 randomized control trials	Alvimopan can accelerate the recovery of GI function (especially for the lower GI tract), shorten the length of hospital stay, and reduce postoperative ileus-related morbidity without compromising opioid analgesia in an enhanced recovery setting.
Nguyen et al. [65]	2015	Alvimopan	Meta-analysis	626 patients from 5 studies	Out of 531 patients undergoing laparoscopic gastrointestinal surgery, patients who were given alvimopan at the standard dose had a 75% relative risk reduction in the development of POI compared to those who were given a placebo.
Al Mazrou et al. [66]	2018	Alvimopan		52,948 patients	Alvimopan, regardless of ileus risk, improves ileus, hospital stay, and ileus-related readmission after intestinal resection, and these effects are sustained over the long term.
Schwenk et al. [67]	2017	Alvimopan, methylnaltrexone, and naloxegol	Systematic review		Peripherally acting μ-opioid receptor antagonists may be effective in treating postoperative ileus, but definitive conclusions are not possible because of study inconsistency and the relatively low quality of evidence. Methylnaltrexone has the most consistent evidence, and its oral formulation may be slightly less effective than the subcutaneous formulation but may cause fewer GI adverse effects.
Lenis et al. [68]	2020	N methylnaltrexone	Retrospective study	29 patients each in control and treatment group	Time to flatus and bowel movement were similar in the group receiving methylnaltrexone vs the group not receiving it.
Gong et al. [69]	2016	Prucalopride (serotonin receptor-5HT4 agonist)	Randomized control trial	55 patients in the placebo group and 55 patients in the treatment group	Prucalopride is a safe and effective treatment to reduce postoperative ileus and systemic inflammation without affecting postoperative complications in patients undergoing elective gastrointestinal surgery.
Daniali et al. [70]	2019	Prucalopride	Systematic review and meta-analysis		Prucalopride can be helpful in the treatment of postoperative ileus; however, the major hurdle is the high cost of the drug.
Stakenborg et al. [47]	2018	Prucalopride	Randomized control trial (RCT)	42 patients were recruited, and 30 completed the study	Preoperative but not postoperative treatment with prucalopride prevents intestinal inflammation and shortens POI in both mice and humans, indicating that preoperative administration of 5-HT4R agonists should be further evaluated as a treatment of POI.
Kram et al. [71]	2018	Neostigmine (cholinergic agonist)	Retrospective observational study	182 patients	Neostigmine administered by the SQ route may be reasonable for the management of ileus.
You et al. [72]	2018	Neostigmine	Randomized control trial (RCT)		ST36 acupoint injection with neostigmine is safe and effective for the treatment of POI.
Petersen et al. [73]	2019	Neostigmine	Case report	-	Neostigmine used in patients may be safe and efficacious for the treatment of refractory ileus in pediatric patients after liver transplantation.
Glowka et al. [74]	2015	Calcitonin gene-related peptide (CGRP)		Mouse subjects	CGRP receptor antagonism could be instrumental in the prevention of POI.
Maehara et al. [75]	2015	5-HT3 receptor antagonists: ondansetron, tropisetron, and palonosetron		Mouse subjects	5-HT3 receptor antagonists restored the delayed gastrointestinal transit by intestinal motility (IM) and should be therapeutically useful agents against POI.
Springer et al. [76]	2018	Simethicone (anti-flatulence)	Randomized control trial (RCT)	118 patients were undergoing colorectal surgery. 58 patients were in treatment vs 60 patients in placebo groups.	This study failed to show a difference in return of gastrointestinal motility in patients receiving simethicone following colorectal surgery vs placebo.
Cooke et al. [77]	2019	Lidocaine	Meta-analysis	405 patients from 9 randomized control trials	Perioperative IV lidocaine may improve the recovery of gastrointestinal function after colorectal surgery.
Moeen et al. [78]	2019	Lidocaine	Randomized control trial (RCT)	111 patients	Between the lidocaine and the control group, mean times to return of bowel sounds, first flatus, first defecation, and resuming of regular diet were significantly shorter in the lidocaine group.
Weibel et al. [79]	2018	IV Lidocaine compared to placebo or no treatment and thoracic epidural analgesia (TEA)	Systematic review	4,525 participants from 68 trials	Uncertain whether lidocaine reduces the risk of ileus, time to first defecation/bowel movement as the quality of evidence was very low for outcomes. The effects of IV lidocaine compared with TEA are unclear of the time to first bowel movement. The risk for ileus was also unclear as only one small trial assessed these outcomes (very low‐quality evidence).
Kranke et al. [80]	2015	Lidocaine vs placebo/no treatment or epidural analgesia	Systematic review	2,802 participants from 45 trials	There is limited evidence that lidocaine, when compared to placebo, had a further impact on gastrointestinal recovery.
Moshiri et al. [81]	2019	Prokinetic agents: metoclopramide and erythromycin	Case report	-	Prokinetics were effective in resolving ileus in a 15-year-old girl who ingested 5 grams methamphetamine (MET).
Bugaev et al. [82]	2019	Prokinetic agents: metoclopramide and erythromycin	Meta-analysis	45 studies	In patients who have undergone abdominal surgery, the study could not recommend for or against the use of either metoclopramide or erythromycin to hasten the resolution of ileus in patients.
Rakowski et al. [83]	2019	Acetaminophen vs ketorolac with patients controlled opioid analgesia pump (PCA)	Randomized control trial (RCT)	100 patients	Use of ketorolac with dilaudid PCA was associated with a quicker return of bowel function than acetaminophen.
Aryaie et al. [84]	2018	Acetaminophen vs placebo with PCA in both groups	Multi-institutional, randomized, double-blinded, placebo-controlled study (DOCIVA study)	100 recruited, 97 included in the study after excluding 3	There appears to be reduced time to return of bowel function and a lower rate of postoperative ileus in patients receiving IV acetaminophen vs placebo.
Burnett et al. [85]	2018	Liposomal bupivacaine (local anesthetic)	Retrospective cohort	61 patients	The use of liposomal bupivacaine in laparotomy patients decreases time to flatus.

## Conclusions

Postoperative ileus is a physiological response of the body due to disruption of bowel motility. Although POI has been reported of significant burden during postoperative recovery, the exact etiology is still not conclusive. It is believed to be mediated by multifactorial causes including neural, inflammatory, hormonal, or pharmacological mechanisms, which are conceptually divided into patient and operative factors, all of which vary during surgical procedures.

Prevention of POI improves recovery and reduces hospital stay. This has been evident with the introduction of enhanced ERAS and minimally invasive surgery. Moreover, standard management of POI includes correction of reversible surgical and medical causes with supportive measures of pain control, intravenous fluid and electrolyte therapy, dietary restriction, and selective placement of a nasogastric tube for gastrointestinal. Therefore, well-established management methods should be introduced across healthcare centers globally, and further studies should be conducted to determine the effectiveness of promising management techniques for postoperative ileus.
